# Synthesis of Gemcitabine-Threonine Amide Prodrug Effective on Pancreatic Cancer Cells with Improved Pharmacokinetic Properties

**DOI:** 10.3390/molecules23102608

**Published:** 2018-10-11

**Authors:** Sungwoo Hong, Zhenghuan Fang, Hoi-Yun Jung, Jin-Ha Yoon, Soon-Sun Hong, Han-Joo Maeng

**Affiliations:** 1 Center for Catalytic Hydrocarbon Functionalization Institute for Basic Science (IBS), Daejeon 34141, Korea; hongorg@kaist.ac.kr (S.H.); spurs9@kaist.ac.kr (H.-Y.J.); 2 Department and of Chemistry, Korea Advanced Institute of Science and Technology (KAIST), Daejeon 34141, Korea; 3 Department of Biomedical Sciences, College of Medicine, Inha University, Incheon 22212, Korea; junghwan110@gmail.com; 4 College of Pharmacy, Gachon University, Incheon 21936, Korea; jinha89@daum.net

**Keywords:** amino acid transporters, amide bond, gemcitabine prodrug, metabolic stability, pancreatic cancer cells, pharmacokinetics

## Abstract

To investigate the amino acid transporter-based prodrug anticancer strategy further, several amino acid-conjugated amide gemcitabine prodrugs were synthesized to target amino acid transporters in pancreatic cancer cells. The structures of the synthesized amino acid-conjugated prodrugs were confirmed by ^1^H-NMR and LC-MS. The pancreatic cancer cells, AsPC1, BxPC-3, PANC-1 and MIAPaCa-2, appeared to overexpress the amino acid transporter LAT-1 by conventional RT-PCR. Among the six amino acid derivatives of gemcitabine, threonine derivative of gemcitabine (Gem-Thr) was more effective than free gemcitabine in the pancreatic cancer cells, BxPC-3 and MIAPaCa-2, respectively, in terms of anti-cancer effects. Furthermore, Gem-Thr was metabolically stable in PBS (pH 7.4), rat plasma and liver microsomal fractions. When Gem-Thr was administered to rats at 4 mg/kg i.v., Gem-Thr was found to be successfully converted to gemcitabine via amide bond cleavage. Moreover, the Gem-Thr showed the increased systemic exposure of formed gemcitabine by 1.83-fold, compared to free gemcitabine treatment, due to the significantly decreased total clearance (0.60 vs. 4.23 mL/min/kg), indicating that the amide prodrug approach improves the metabolic stability of gemcitabine in vivo. Taken together, the amino acid transporter-targeting gemcitabine prodrug, Gem-Thr, was found to be effective on pancreatic cancer cells and to offer an efficient potential means of treating pancreatic cancer with significantly better pharmacokinetic characteristics than gemcitabine.

## 1. Introduction

Gemcitabine (2′-2′-difluorodeoxycytidine, dFdC) is a standard anticancer agent that is used to treat all stages of pancreatic adenocarcinoma [1], and combinations of gemcitabine with other anti-cancer agents, such as erlotinib [2], nab-paclitaxel [3,4] and others have been applied to pancreatic cancer patients. Because gemcitabine has serious toxic effects on the gastrointestinal track [5], and low oral bioavailability (BA), it is administered intravenously [6]. However, gemcitabine is a metabolically unstable drug and has a half-life of only 9 min in man, probably due to its rapid conversion to 2′-2′-difluorodeoxyuridine (dFdU) by cytidine deaminase (CD), which is abundant in blood and tissues [7]. This poor metabolic stability and poor compliance has created a clinical need for a gemcitabine prodrug. Several types of prodrugs have been reported, such as valproate amide [8,9], amino acid ester [10,11], aminoacyl amide derivative [12], and l-carnitine amide (OCTN2 targeting) [13] prodrugs.

To maintain the growth and survival of cancer cells during neoplastic transformation, amino acid production is massively increased and as a result, several uptake transporters (e.g., amino acid transporters) are overexpressed. For example, l-type amino acid transporter 1 (LAT-1), a representative amino acid transporter, is overexpressed in various types of cancer cells [14], and interestingly, LAT-1 expression has been consistently reported to be elevated in patients with pancreatic cancer and in transplanted Colo357 cells (a pancreatic cancer cell line) [15,16]. Accordingly, LAT-1 has been suggested to be a versatile target for the development of transporter-based drugs [14].

Our research group have been investigating the merits of strategies based on the amino acid transporter-based prodrug approach to overcome the multidrug resistance (MDR) triggered by various efflux transporters like P-glycoprotein (P-gp, MDR1) in cancer cells [17,18,19]. For example, the addition of amino acids, such as, l-valine (Val) or tyrosine (Tyr), to lapatinib by amide bond formation successfully enhanced the anti-cancer effect of lapatinib in human breast cancer cells (MDA-MB-231 and MCF7) and lung cancer cells (A549), without plasma stability issues, which demonstrated the involvements of amino acid transporters [18]. More recently, the val-modified amide prodrug of doxorubicin (Dox-Val) improved cellular uptake efficiency in MCF7 cells [19]. However, in a rat pharmacokinetic study that demonstrated the conversion of Dox-val to Dox by amide bond cleavage in vivo, the pharmacokinetic properties of the amino acid prodrug examined were limited [19].

Based on the observation that amino acid transporters (e.g., LAT-1) are overexpressed in various cancer cells for their survival, we speculated that amino acid conjugated prodrug of gemcitabine might be an effective way to improve gemcitabine uptake by overexpressing amino acid transporters in pancreatic cancer cells as well as overall physicochemical property. In the present study, we successfully synthesized a threonine-gemcitabine (Gem-Thr) prodrug and investigated its anti-cancer efficacy in pancreatic cancer cells and its in vitro/in vivo metabolic stabilities. In addition, we examined LAT-1 (an amino acid transporter) mRNA/protein expressions in pancreatic cancer cells.

## 2. Results and Discussion

### 2.1. Synthesis of Gemcitabine Prodrugs with Amino Acids

**Figure 1:**
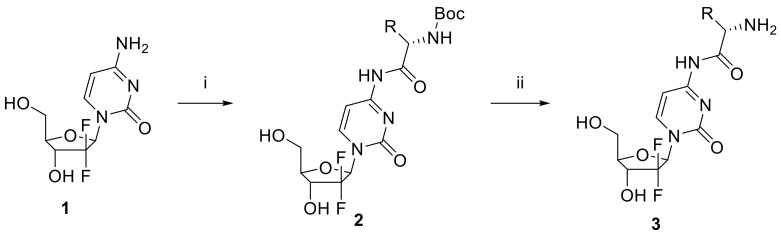
Synthetic scheme of the procedure used to synthesize gemcitabine-amino acid prodrugs. Reagent and conditions were as follows; (i) *N*-Boc-l-amino acid, 4-methylmorpholine, HOBt, EDCI·HCl, DMF/DMSO = 3: 1, 55 °C, 17 h; (ii) 4N HCl in dioxane, dry CH_2_Cl_2_, r.t. 30 min.

To confirm the validity of the amino acid transporter-based prodrug approach for gemcitabine, we prepared a series of amino acid conjugated gemcitabine prodrugs. As summarized in Figure 1, gemcitabine-amino acid prodrugs **3** were obtained from gemcitabine (**1**) using a two-step sequence, that is by amide bond formation between gemcitabine (**1**) and *N*-Boc-l-amino acid employing 1-hydroxy-1H-benzotriazole (HOBt), 1-ethyl-3-(3-dimethylaminopropyl)carbodiimide and 4-methylmorpholine in DMF/DMSO (3:1), and subsequent Boc deprotection of the resulting compound **2** using hydrochloric acid. In order to avoid potential problems associated with epimerization when using carbodiimides as coupling reagents, HOBt was used as an additive because it enhances the reactivities of activated ester intermediates by facilitating amine approach via hydrogen bonding. To confirm the formation of amide bond, an intermediate of representative compound (Gem-Thr-Boc) was dissolved in DMSO-*d*_6_. In gemcitabine part, the chemical shift of CH(5) in oxopyrimidine core showed at *δ* 7.26 (*J*_5,6_ = 7.6 Hz) as a doublet by *ortho* coupling with CH(6) at *δ* 8.27. The characteristic amide NH peak was detected at *δ* 10.99 as a broad singlet, and Boc group of amino acid was appeared at *δ* 1.39. The side chain of threonine CH_3_ was appeared at *δ* 1.39 having a coupling constant *J* = 6.3 Hz and OH group was resonated at *δ* 4.89. In addition, the MS/MS spectrum showed [M + H]^+^ at *m*/*z* 465.2, which confirmed the chemical bond between gemcitabine and *N*-Boc protected threonine. The *N*-Boc of amino acid was deprotected in acidic conditions to afford the final Gem-Thr product that was confirmed by ^1^H NMR in CD_3_OD. The peak of Boc group disappeared and concurrently the side chain of threonine CH_3_ was detected as a doublet peak at *δ* 7.26 (*J* = 6.4 Hz). Furthermore, CH(5)- and CH(6)-peaks of oxopyrimidine remained at *δ* 6.11 and *δ* 7.85 coupled as a doublet (*J*_5,6_ = 7.6).

### 2.2. Expression of LAT-1 in Pancreatic Cancer Cell Lines

**Figure 2:**
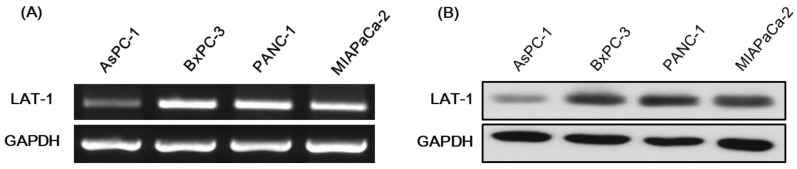
mRNA (**A**) and protein (**B**) expressions of LAT-1 in various pancreatic cancer cell lines (AsPC-1, BxPC-3, PANC-1, and MIAPaCa-2). Reverse transcription–PCR and Western blot were performed using gel electrophoresis after purification of DNA from cancer cells.

LAT-1 mRNA and protein levels were detected in the pancreatic cancer cell lines AsPC1, BxPC-3, PANC-1 and MIAPaCa-2. LAT-1 expression was greatest in BxPC-3 (Figure 2). The following studies on anti-cancer effects were performed using BxPC-3 and MIAPaCa-2 cells. Therefore, this transcriptional activation of LAT-1 might expect to facilitate transport capability of amino acid linked gemcitabine.

### 2.3. Anticancer Effects of Prodrugs with Amino Acids in Cancer Cells

**Figure 3:**
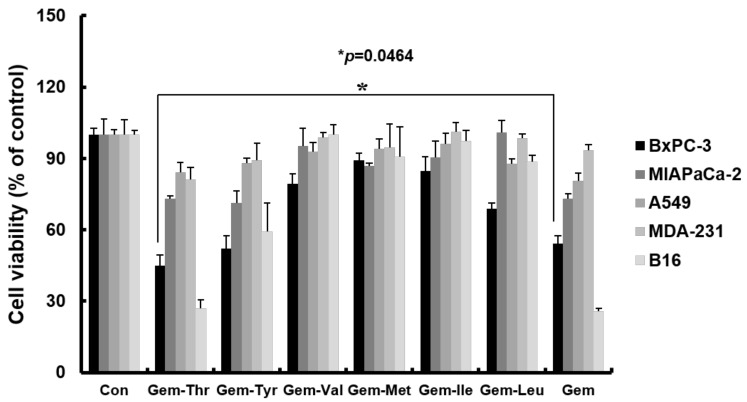
Growth-inhibitory effects of gemcitabine and of its amino-acid-conjugated derivatives on BxPC-3, MIAPaCa-2, A549, MDA-MB-231 B16 cells after exposure to 1 μM concentrations for 48 h, as estimated by MTT assay (mean ± SD, *n* = 3). Experiments were performed three times independently. * *p* < 0.05, compared with free gemcitabine group.

We evaluated the cytotoxic effects of gemcitabine derivatives on various cancer cell lines including PDAC (pancreatic ductal adenocarcinoma), and compared results with those of free gemcitabine (Figure 3). In A549 (lung cancer cells) and MDA-MB-231 (breast cancer cells) neither gemcitabine nor gemcitabine derivatives had any significant anti-cancer effect versus vehicle treated controls. However, gemcitabine had significant anti-cancer effects on BxPC-3, MIAPaCa-2 (pancreatic cancer cells) and B16 (melanoma cells). The anti-cancer effects of Gem-Tyr, Gem-Val, Gem-Met, Gem-Ile and Gem-Leu were similar to those of gemcitabine in pancreatic cancer cells, whereas Gem-Thr had the most potent anticancer effect, and this was slightly superior to that of gemcitabine in BxPC-3 cells (Gem-Thr, 44.7% vs. gemcitabine, 54.1% of cell viability, *p* = 0.0464), which was found to overexpress amino acid transporters, including LAT-1 (Figure 3). The introduction of threonine to gemcitabine is likely to be recognized by the LAT-1, which has an important role to influx the drug into the cancer cells. However, addition of some amino acid moieties to gemcitabine did not exert the enhanced cytotoxic effects on the pancreatic cancer cells. Similarly, a valine ester prodrug (Val-SN-38) showed a comparable cytotoxic effect compared to SN-38, an active metabolite of irinotecan, despite an increased intracellular accumulation in MCF cells with amino acid transporters being overexpressed [17].

**Figure 4:**
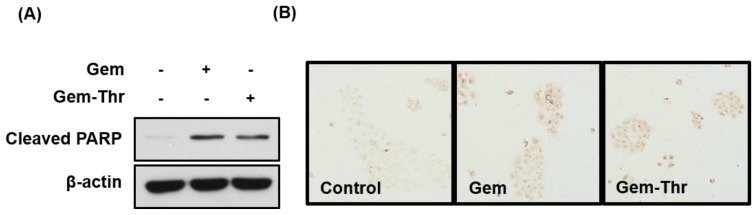
Apoptosis of pancreatic cancer cells by Gemcitabine derivative. After the treatment of gemcitabine and Gem-Thr to BxPC-3 cells for 24 h, cleaved PARP was detected by western blotting (**A**) and TUNEL assay (**B**) was performed.

Additionally, consistent with MTT results, either gemcitabine or Gem-Thr induced apoptosis significantly when we measured cleaved PARP as well as TUNEL positive cells after a 24 h treatment (Figure 4). 

### 2.4. In Vitro Plasma Stability of Gem-Thr

**Figure 5:**
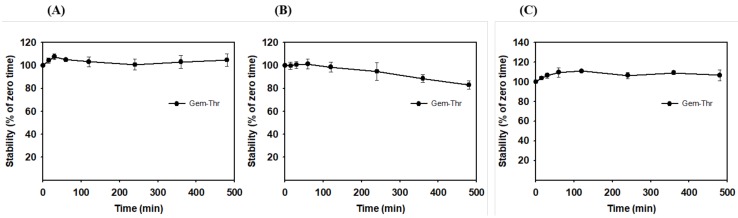
In vitro metabolic stabilities of Gem-Thr in PBS (**A**), plasma (**B**) and a liver microsome fraction (**C**) (mean ± SD, *n* = 3).

To recognize a substrate by amino acid transporters in cancer cells, the stability of the amide prodrug with amino acid moiety, Gem-Thr, should be first possessed [18]. Thus, the stability of the prodrug was investigated in PBS, rat plasma and liver microsomes for up to 8 h (Figure 5). Gem-Thr was metabolically stable in PBS (pH 7.4), whereas 20% of Gem-Thr was metabolized in rat plasma after 8 h, indicating Gem-Thr can be metabolized to gemcitabine by enzymes in plasma. Surprisingly, Gem-Thr was metabolically stable in rat liver microsomal fractions, which contain high levels of cytochrome P450 (CYP) enzymes, indicating the amide bond between gemcitabine and threonine cannot be readily broken via phase I metabolism or enzymes expressed in liver microsomes. This concurs with the results of a previous study on val-lapatinib and tyr-lapatinib, in which amide linkage was found to result in appropriate metabolic stability in vitro [18]. Our results indicate the amino acid moiety of Gem-Thr is likely both stable and recognized by the amino acid transporters in cancer cells.

### 2.5. Comparison of Systemic Pharmacokinetics with Free Gemcitabine

**Figure 6:**
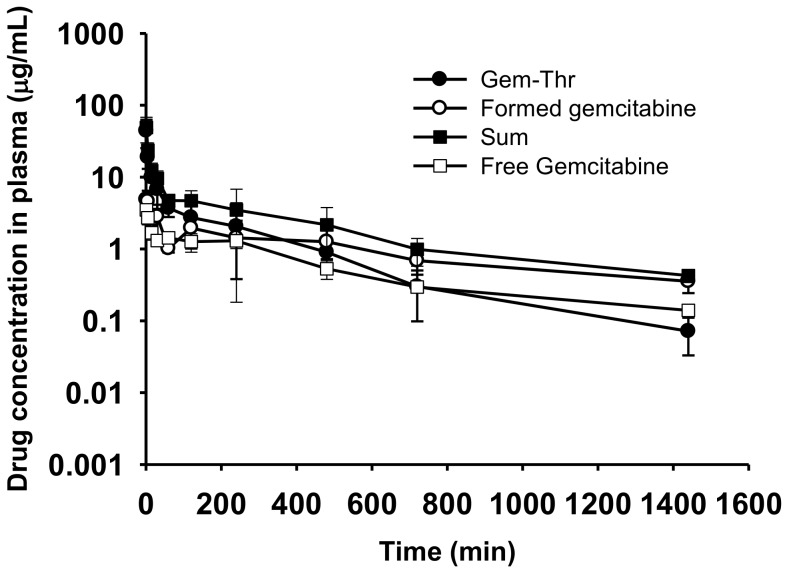
Plasma concentration-time profiles of Gem-Thr (prodrug), formed gemcitabine (metabolite), the sum of Gem-Thr and formed gemcitabine and free gemcitabine. The intravenous pharmacokinetics of Gem-Thr was compared to that of free gemcitabine (4 mg/kg, *n* = 4).

The pharmacokinetic parameters of Gem-Thr and free gemcitabine after intravenous administration in rats are summarized in Table 1. Concentrations of Gem-Thr and gemcitabine and their summed concentrations in plasma were measured and compared to those of animals administered free gemcitabine (Figure 6). After gemcitabine administration at 4 mg/kg, the oral systemic exposure (i.e., AUC) and the measure of drug elimination form the body, CL, were found to be 948.38 ± 52.04 μg∙min/mL and 4.23 ± 0.23 mL/min/kg, respectively, which concurred with previously reported values [20]. The volume of distribution at steady state (V_ss_) and average time for a drug molecule to reside in the body, MRT, values for gemcitabine were 2483.64 ± 867.19 mL/kg and 582.06 ± 177.90 min (Table 1). After administration of Gem-Thr at 4 mg/kg i.v., the conversion of Gem-Thr to gemcitabine was found to be probably due to amide bond cleavage, with similar systemic exposure (i.e., AUC) between Gem-Thr and the formed gemcitabine. More importantly, Gem-Thr increased systemic exposure (i.e., the AUC of gemcitabine) by 1.83-fold versus free gemcitabine, and this was attributed to a significantly lower total CL value (0.60 vs. 4.23 mL/min/kg) (Table 1). Furthermore, this suggests the amide prodrug approach improves the metabolic stability of gemcitabine in vivo. Other pharmacokinetic parameters, such as, V_ss_ and MRT, were also found to be different for Gem-Thr and gemcitabine (Table 1). For example, MRT of gemcitabine for Gem-Thr was greater than that for gemcitabine, indicating that formed gemcitabine from Gem-Thr remains in the systemic circulation longer than free gemcitabine. In a previous study, an amide prodrug of gemcitabine releasing gemcitabine and valproic acid was found to enhance gemcitabine stability by blocking its deamination to uridine, and to prolong systemic exposure as compared with gemcitabine alone [8]. Furthermore, the AUC of sum of Gem-Thr and the gemcitabine (metabolite) was significantly higher (3.62-fold higher) than gemcitabine alone (Table 1). In our previous study, although we first demonstrated the successful conversion and systemic circulation of the active metabolite (doxorubicin) probably occurred due to amide bond cleavage, amide linked doxorubicin/valine failed to improve pharmacokinetic properties including systemic CL [19]. On the other hand, Gem-Thr showed enhanced gemcitabine stability in vivo and an increased AUC and decreased CL versus gemcitabine. This enhanced systemic stability of Gem-Thr is likely to improve gemcitabine uptake by cancer cells and to target amino acid transporters like LAT-1 transporters.

## 3. Materials and Methods

### 3.1. Materials

Commercial grade reagents and solvents were used without further purification. Thin layer chromatography (TLC) was performed using precoated silica gel 60 F^254^ plates and visualized using anisaldehyde solution, heat, and UV light (254 nm). Flash column chromatography was undertaken on silica gel (400–630 mesh). ^1^H NMR was recorded at 400 MHz and chemical shifts are quoted in parts per million (ppm) versus an appropriate solvent peak or 2.50 ppm for DMSO-*d*_6_. The following abbreviations were used to describe peak splitting patterns: br = broad, s = singlet, d = doublet, t = triplet, q = quartet, m = multiplet, dd = doublet of doublets, td = triplet of doublets, ddd = doublet of doublets of doublets. Coupling constants, *J*, are reported in hertz (Hz). HPLC was conducted using an Agilent HPLC unit equipped with an Agilent Poroshell 120 EC-C18 reverse phase column (4.6 × 50 mm, 2.7 Micron) and mass spectroscopy was performed using a quadrupole LC/MS unit.

### 3.2. Synthesis and Characterization of DOX-Val

#### 3.2.1. General Procedure for Preparing Gemcitabine Derivatives 

To a solution of gemcitabine (1.0 equiv), 1-ethyl-3-(3-dimethylaminopropyl)-carbodiimide hydrochloride (1.3 equiv), 4-methylmorpholine (1 equiv), and 1-hydroxybenzotriazole (1 equiv) in DMF/DMSO (5 mL, 3:1) was added dropwise *N*-Boc protected amino acid (1.1 equiv) at room temperature in a N_2_ atmosphere. The reaction mixture was then stirred in an oil bath at 55 °C for 17 h, cooled to room temperature and quenched by adding brine (5 mL). The mixture was then extracted using ethyl acetate (3 × 10 mL) and the combined organic layer was washed with 20% LiCl solution, saturated NaHCO_3_ aqueous solution, and brine, dried over MgSO_4_, and concentrated under reduced pressure. The residue was purified by silica gel column chromatography (DCM/methanol = 15:1) to afford the desired product **2**. To a solution of the mixture of aforementioned intermediate in anhydrous DCM (1 mL) was added 4N HCl in dioxane (1 mL). The mixture was then stirred for 12 h at room temperature, solvent was removed, and the residue was purified by silica gel flash column chromatography (DCM/methanol = 3:1) to afford the desired product **3**.

#### 3.2.2. (*S*)-2-Amino-*N*-(1-((2*R*,4*R*,5*R*)-3,3-difluoro-4-hydroxy-5-(hydroxymethyl)tetrahydrofuran-2-yl)-2-oxo-1,2-dihydropyrimidin-4-yl)-3-methylbutanamide (Gem-Val)

From the reaction of gemcitabine (27 mg, 0.104 mmol), 21 mg (56% for 2 steps) was obtained. ^1^H NMR (400 MHz, Methanol-*d*_4_) δ 7.81 (d, *J* = 7.6 Hz, 1H), 6.21 (t, *J* = 7.9 Hz, 1H), 6.04 (d, *J* = 7.6 Hz, 1H), 4.51 (d, *J* = 7.5 Hz, 1H), 4.25 (td, *J* = 12.1, 8.3 Hz, 1H), 3.94 (dd, *J* = 12.7, 2.5 Hz, 1H), 3.88 (dt, *J* = 8.4, 2.9 Hz, 1H), 3.78 (dd, *J* = 12.5, 3.2 Hz, 1H), 2.14 (h, *J* = 6.8 Hz, 1H), 1.00 (dd, *J* = 6.8, 5.5 Hz, 6H); MS/MS *m*/*z* 363.1 (M + 1)^+^.

#### 3.2.3. (2*S*,3*R*)-2-Amino-*N*-(1-((2*R*,4*R*,5*R*)-3,3-difluoro-4-hydroxy-5-(hydroxylmethyl)tetra-hydrofuran-2-yl)-2-oxo-1,2-dihydropyrimidin-4-yl)-3-hydroxybutanamide2-(4-((pyridin-3-ylmethyl)amino)quinazolin-2-yl)phenol (Gem-Thr)

From the reaction of gemcitabine (51 mg, 0.194 mmol), 24 mg (34%) was obtained. ^1^H NMR (400 MHz, Methanol-*d*_4_) δ 7.85 (d, *J* = 7.6 Hz, 1H), 6.25–6.16 (m, 1H), 6.11 (d, *J* = 7.6 Hz, 1H), 4.67 (d, *J* = 3.9 Hz, 1H), 4.32–4.19 (m, 2H), 3.98–3.92 (m, 1H), 3.89 (dt, *J* = 8.4, 2.9 Hz, 1H), 3.79 (dd, *J* = 12.6, 3.3 Hz, 1H), 1.21 (d, *J* = 6.4 Hz, 3H); MS/MS *m*/*z* 365.1 (M + 1)^+^.

(Gem-Thr-Boc intermediate) ^1^H NMR (400 MHz, DMSO-*d*_6_) δ 10.99 (s, 1H), 8.27 (d, *J* = 7.6 Hz, 1H), 7.26 (d, *J* = 7.6 Hz, 1H), 6.49 (d, *J* = 8.7 Hz, 1H), 6.32 (d, *J* = 6.4 Hz, 1H), 6.18 (t, *J* = 7.4 Hz, 1H), 5.31 (t, *J* = 5.5 Hz, 1H), 4.89 (s, 1H), 4.25–4.11 (m, 2H), 4.08–4.00 (m, 1H), 3.89 (dt, *J* = 8.5, 3.0 Hz, 1H), 3.85–3.77 (m, 1H), 3.65 (ddd, *J* = 12.8, 6.0, 3.2 Hz, 1H), 1.39 (s, 8H), 1.09 (d, *J* = 6.3 Hz, 3H); MS/MS *m*/*z* 465.1 (M + 1)^+^.

#### 3.2.4. (*S*)-2-Amino-*N*-(1-((2*R*,4*R*,5*R*)-3,3-difluoro-4-hydroxy-5-(hydroxymethyl)tetrahydrofuran-2-yl)-2-oxo-1,2-dihydropyrimidin-4-yl)-3-(4-hydroxyphenyl)propanamide (Gem-Tyr)

From the reaction of gemcitabine (22 mg, 0.083 mmol), 27 mg (76%) was obtained. ^1^H NMR (400 MHz, Methanol-*d*_4_) δ 7.77 (d, *J* = 7.6 Hz, 1H), 7.08 (d, *J* = 8.5 Hz, 2H), 6.68 (d, *J* = 8.5 Hz, 2H), 6.25–6.13 (m, 1H), 5.93 (d, *J* = 7.6 Hz, 1H), 4.23 (td, *J* = 12.1, 8.2 Hz, 1H), 3.96–3.90 (m, 1H), 3.87 (dt, *J* = 8.4, 2.9 Hz, 1H), 3.77 (dd, *J* = 12.6, 3.2 Hz, 1H), 3.09 (dd, *J* = 13.9, 6.3 Hz, 1H), 2.88 (dd, *J* = 13.8, 8.2 Hz, 1H); MS/MS *m*/*z* 427.1 (M + 1)^+^.

#### 3.2.5. (*S*)-2-Amino-*N*-(1-((2*R*,4*R*,5*R*)-3,3-difluoro-4-hydroxy-5-(hydroxymethyl)tetrahydrofuran-2-yl)-2-oxo-1,2-dihydropyrimidin-4-yl)-4-(methylthio)butanamide (Gem-Met)

From the reaction of gemcitabine (38 mg, 0.146 mmol), 23 mg (40%) was obtained. ^1^H NMR (400 MHz, Methanol-*d*_4_) δ 7.83 (d, *J* = 7.7 Hz, 1H), 6.34–6.13 (m, 1H), 5.99 (d, *J* = 7.6 Hz, 1H), 4.83–4.73 (m, 1H), 4.25 (td, *J* = 12.1, 8.2 Hz, 1H), 3.98–3.91 (m, 1H), 3.89 (dt, *J* = 8.4, 2.8 Hz, 1H), 3.78 (dd, *J* = 12.5, 3.2 Hz, 1H), 2.55 (ddd, *J* = 8.4, 6.4, 3.7 Hz, 2H), 2.23–2.11 (m, 1H), 2.09 (s, 3H), 2.07–1.89 (m, 1H); MS/MS *m*/*z* 395.1 (M + 1)^+^.

#### 3.2.6. (2*S*,3*S*)-2-Amino-*N*-(1-((2*R*,4*R*,5*R*)-3,3-difluoro-4-hydroxy-5-(hydroxymethyl)tetrahydrofuran-2-yl)-2-oxo-1,2-dihydropyrimidin-4-yl)-3-methylpentanamide (Gem-Ile)

From the reaction of gemcitabine (29 mg, 0.110 mmol), 20 mg (50%) was obtained. ^1^H NMR (400 MHz, Methanol-*d*_4_) δ 7.81 (d, *J* = 7.6 Hz, 1H), 6.28–6.14 (m, 1H), 6.02 (d, *J* = 7.6 Hz, 1H), 4.54 (d, *J* = 8.1 Hz, 1H), 4.25 (td, *J* = 12.0, 8.2 Hz, 1H), 3.98–3.89 (m, 1H), 3.88 (dt, *J* = 8.4, 2.9 Hz, 1H), 3.78 (dd, *J* = 12.5, 3.3 Hz, 1H), 1.97–1.83 (m, 1H), 1.58 (ddd, *J* = 13.6, 7.6, 3.4 Hz, 1H), 1.32–1.15 (m, 1H), 0.99 (d, *J* = 6.8 Hz, 3H), 0.92 (t, *J* = 7.4 Hz, 3H); MS/MS *m*/*z* 377.2 (M + 1)^+^.

#### 3.2.7. (*S*)-2-Amino-*N*-(1-((2*R*,4*R*,5*R*)-3,3-difluoro-4-hydroxy-5-(hydroxymethyl)tetrahydrofuran-2-yl)-2-oxo-1,2-dihydropyrimidin-4-yl)-4-methylpentanamide (Gem-Leu)

From the reaction of gemcitabine (19 mg, 0.073 mmol), 21 mg (77%) was obtained. ^1^H NMR (400 MHz, Methanol-*d*_4_) δ 7.81 (d, *J* = 7.6 Hz, 1H), 6.26–6.15 (m, 1H), 5.97 (d, *J* = 7.6 Hz, 1H), 4.75–4.68 (m, 1H), 4.25 (td, *J* = 12.1, 8.2 Hz, 1H), 3.98–3.91 (m, 1H), 3.88 (dt, *J* = 8.3, 2.8 Hz, 1H), 3.78 (dd, *J* = 12.5, 3.2 Hz, 1H), 1.77–1.56 (m, 3H), 0.98 (d, *J* = 6.1 Hz, 3H), 0.95 (d, *J* = 6.1 Hz, 3H); MS/MS *m*/*z* 377.2 (M + 1)^+^.

### 3.3. Characterization of Gemcitabine Prodrugs with Amino Acid

#### 3.3.1. Cell Culture

Human pancreatic cancer cells (MIAPaCa-2, BxPC-3 and AsPC-1) [21], lung cancer cell lines (A549) [22], human breast cancer cell lines (MDA-MB-231) [23] and melanoma cell lines (B16) [24] were purchased from the American Type Culture Collection (Manassas, VA, USA). MIAPaCa-2 and B16-F10 cells were cultured in Dulbecco’s modified Eagle’s medium (DMEM) supplemented with 10% heat-inactivated fetal bovine serum (FBS) and 1% penicillin/streptomycin. Aspc-1, BxPC-3 MDA-MB-231 and A549 cells were cultured in Roswell Park Memorial Institute 1640 (RPMI-1640) medium supplemented with 10% FBS and 1% penicillin/streptomycin. FBS and all other reagents used for cell culture were purchased from Invitrogen (Carlsbad, CA, USA). Cultures were maintained at 37 °C in 95% air /5% CO_2_ humidified atmosphere. 

#### 3.3.2. Reverse Transcription-PCR

Total LAT-1 RNA was isolated using Trizol reagent and subjected to reverse transcription-PCR (Promega Corp.). The PCR primers used were LAT1, 5′-CCTCTGGGCCTGTTCTCTTG-3′ (forward) and 5′-CTTGAGGCATGTCCACCTCC-3′ (reverse). PCR reaction of forward and reverse genes was performed using the Ex Taq DNA Polymerase Recombinant (TaKaRa, Tokyo, Japan), with final concentrations of 1X PCR buffer, 2.5 mM of dNTP mixture, 2.5 mM of MgCl_2_, 10 pmol of each primer in a total reaction volume of 25 µL containing 1 µL of cDNA. Individual PCR amplification cycle of forward or reverse genes was performed with an initial denaturation step at 94 °C for 3 min, followed by 35 cycles (94 °C for 30 s; 55 °C for 60 s; 72 °C for 60 s), and finally with an elongation step at 72 °C for 5 min. The DNA products were resolved using gel electrophoresis (1.5% agarose gel).

#### 3.3.3. Western Blot Assays

BxPC-3 cells were washed with DPBS and lysed with RIPA buffer (Biosesang, Seongnam, Korea) containing 150 mM NaCl, 1% Triton X-100, 1% sodium deoxycholate, 0.1% SDS, 50 mM Tris-HCl (pH 7.5), 2 mM EDTA (pH 8.0), and Xpert protease inhibitor and phosphatase inhibitor Cocktail (GenDEPOT, Barker, TX, USA). Proteins were separated by 8% or 15% SDS-PAGE (sodium dodecylsulfate-polyacrylamide gel electrophoresis) and transferred to polyvinylidene fluoride (PVDF) membranes. Protein transfer was confirmed using a Ponceau S staining solution (AMRESCO, Solon, OH, USA), and the blots were then immunostained with appropriate primary antibodies (1:1000) and secondary antibodies (1:5000) conjugated to horseradish peroxidase (HRP). Antibody binding was detected using an enhanced chemiluminescence (ECL) reagent (Bio-Rad, Hercules, CA, USA) using primary antibodies specific to proteins of interest, and proteins were detected using X-ray film and enhanced chemiluminescence reagent. Primary antibodies against the following were used: LAT-1, cleaved PARP (Cell Signaling Technologies, Beverly, MA, USA), GAPDH (Abcam, Cambridge, MA, USA), and β-actin (Sigma Aldrich, St. Louis, MO, USA). Secondary antibodies were purchased from Cell signaling technology.

#### 3.3.4. Cytotoxicity Assay in Pancreatic Cells

Cancer cell viabilities after treatment with gemcitabine or its derivatives were quantified using MTT assay as described previously [25]. Briefly, cells were seeded onto 96-well plates at 3 × 10^3^ cells per well and incubated at 37 °C. The cells were treated with each compound at indicated concentrations for 48 h and then, 20 μL of MTT labeling mixture was added to each well. After incubation for 4 h, optical densities (OD) were determined using a microplate reader by measuring absorbances at 540 nm.

#### 3.3.5. Terminal Deoxynucleotidyl Transferase–Mediated Nick End Labeling (TUNEL) Assay 

TUNEL assay was conducted with the ApopTag^®^ Peroxidase In Situ Apoptosis Detection Kit (Merck Millipore, Burlington, MA, USA). Briefly, BxPC-3 cells were seeded onto 18-mm cover glasses in medium and grown to ~70% confluence over 24 h. Cells were then treated with 1 µM of gemcitabine or Gem-Thr for 24 h, fixed in an ice-cold mixture of acetic acid and ethanol solution, washed with DPBS, TUNEL stained, mounted, and examined under a light microscope for nuclear fragmentation. 

### 3.4. In Vitro Metabolic Stability of Gem-Thr

The metabolic stability of Gem-Thr was examined in the presence of PBS (pH 7.4), rat plasma, or liver microsomes as described previously (Maeng et al., 2014). Fresh rat plasma was obtained from sacrificed SD rats (260–280 g) after centrifuging blood (12,000× *g*, 15 min). Rat liver microsomes were obtained from BD Gentest. Gem-Thr (10 µM) was spiked into PBS, blank rat plasma, or liver microsomes (1 mg protein/mL) and incubated in a shaking water bath at 37 °C for 8 h. Aliquots (50-µL) were taken after 0, 15, 30, 60, 120, 240, 260, or 480 min of incubation, immediately pretreated with ice-cold methanolic solution including the internal standard, vortexed for 3 min and centrifuged (12,000× *g*, 10 min). Supernatants were stored at −80 °C until required for analysis.

### 3.5. Systemic Pharmacokinetics Study of Gem-Thr in Rats

This study was performed in male Sprague-Dawley (SD) rats (Orient Bio, Sungnam, Korea), as described previously [26]. Rats were provided water and food *ad libitum* and maintained under a 12:12-h light/dark cycle. On the experimental day, a femoral vein and contralateral artery were cannulated using an Intramedic™ polyethylene tube (PE-50; Becton Dickinson Diagnostics, Sparks, MD, USA) under Zoletil induced anesthesia (50 mg/kg intramuscularly; Virbac, Carros, France). For the intravenous pharmacokinetic study, Gem-Thr or gemcitabine solution were injected into a femoral vein at 4 mg/kg. Blood (~0.22 mL) was taken from the femoral artery at 0 (blank), 1, 5, 15, 30, 60, 120, 240, 480, 720, or 1440 min. To prevent blood loss, the same volume of normal saline was injected intravenously at each time point. Plasma was obtained immediately centrifugation and then stored at −80 °C prior to LC-MS/MS. 

The pharmacokinetic parameters of Gem-Thr, gemcitabine, Gem-Thr plus gemcitabine, and free gemcitabine were calculated by non-compartmental analysis using WinNonlin (Version 3.1; Pharsight, Mountain View, CA, USA), as described previously (Park et al., 2016).

### 3.6. Analysis of Gem-Thr and Gemcitabine by LC-MS/MS

To determine concentrations of Gem-Thr and gemcitabine in rat plasma, plasma samples were deproteinized by adding two volumes of acetonitrile containing internal standard (phenacetin). After mixing, mixtures were centrifuged at 14,000× *g* for 10 min. 

Aliquots of supernatants (2-µL) were injected into the LC-MS/MS system, which consisted of an Agilent HPLC and an Agilent 6490 QQQ mass spectrometer equipped with an ESI+ Agilent Jet Stream ion source (Agilent Technologies, Santa Clara, CA, USA). The separation of each drug and IS from endogenous plasma substances was achieved on a Synergi Polar-RP 80A column (150 × 2.0 mm, 4 μm; Phenomenex, Torrance, CA, USA). The mobile phase consisted of 0.1% formic acid and acetonitrile (20:80, *v*/*v*) at a flow rate of 0.2 mL/min. The column and autosampler tray were maintained at 25 and 4 °C, respectively. Gemcitabine, Gem-Thr, and the IS were quantified by multiple-reaction monitoring (MRM) in positive electrospray ionization mode. Respective precursor-to-product ion transitions were as follows: Gemcitabine, 264.1→112.1, Gem-Thr, 387.1→343.2, and IS (phenacetin), 180.2→162.2. Data acquisition was performed using Mass Hunter software (ver. A.02.00; Agilent Technologies).

### 3.7. Statistical Analysis

Results are expressed as means ± standard deviations (SDs). Differences between group means were analyzed using the two-tailed Student’s *t*-test. Statistical significance was accepted for *p* values < 0.05.

## 4. Conclusions

Various amino acid derivatives of gemcitabine were successfully synthesized by forming amide bonds. Of the six derivatives of gemcitabine synthesized, Gem-Thr most effectively killed pancreatic cancer cells, in which LAT-1 was overexpressed. Our in vitro metabolic stability showed Gem-Thr is stable in PBS, plasma and a liver microsomal fraction, which demonstrated Gem-Thr is stable in cancer cells overexpressing amino acid transporter. Interestingly, our systemic pharmacokinetic results suggested that the amide prodrug approach improves the metabolic stability of gemcitabine in our in vivo model due to reduced decreased metabolic clearance. To the best of our knowledge, this is the first report on the cytotoxic effects of an amino acid transporter-targeting gemcitabine prodrug, produced by the introduction of threonine, on pancreatic cancer cells. Although the in vitro anti-cancer effect of Gem-Thr was only slightly superior to that of free gemcitabine, this improved pharmacokinetic property of Gem-Thr may have substantial anti-cancer effects in pancreatic cancer.

## Figures and Tables

**Table 1:** Comparison of pharmacokinetic parameters of Gem-Thr, formed gemcitabine and sum of two species after intravenous administration of Gem-Thr at a dose of 4 mg/kg with those of free gemcitabine alone at a dose of 4 mg/kg in rats (*n* = 3–4).

Pharmacokinetic Parameters	Gem-Thr (4 mg/kg)			Free Gemcitabine (4 mg/kg)
	Gem-Thr	Gemcitabine	Sum	
AUC (μg∙min/mL)	1713.85 ± 1082.40	1739.88 ± 282.00 *	3437.92 ± 1180.56	948.38 ± 52.04
Terminal t_1/2_ (min)	236.18 ± 50.94	666.83 ± 271.49	537.23 ± 227.78	532.68 ± 177.90
CL (mL/min/kg)	2.85 ± 1.33	0.60 ± 0.10 *	1.26 ± 0.39	4.23 ± 0.23
V_ss_ (mL/kg)	662.35 ± 281.40	545.57 ± 263.01 *	770.96 ± 435.31	2483.64 ± 867.19
MRT (min)	237.18 ± 20.87	907.18 ± 391.68 *	577.36 ± 212.90	582.06 ± 177.90

Administration of dose for Gem-thr was 4 mg/kg, which is equivalent to 2.892 mg/kg for gemcitabine. Data presents as mean ± standard deviation (SD). * Significantly different from free gemcitabine group (*p* < 0.05).
